# Acute Hyperhidrosis: A Clue to Underlying Autonomic Dysfunction and a Rare Neurological Disorder

**DOI:** 10.7759/cureus.76387

**Published:** 2024-12-25

**Authors:** Rachel Peh, Chun Wai Yip, Zhong Hong Liew

**Affiliations:** 1 Internal Medicine, Singapore General Hospital, Singapore, SGP; 2 Neurology, Singapore General Hospital, Singapore, SGP

**Keywords:** autoimmune encephalitis, autonomic nervous system dysfunction, generalized hyperhidrosis, lgi1 antibody autoimmune encephalitis, secondary hyperhidrosis

## Abstract

Acute hyperhidrosis is characterized by excessive sweating. In the absence of other symptoms, the symptoms of sweating alone are often benign and may be ignored by patients and clinicians. Rarely, hyperhidrosis may be a harbinger of an underlying severe disease. Autonomic nervous system dysfunction leading to hyperactivity of the sympathetic nervous system can result in excessive sweating. This case report is about a gentleman who presented with acute hyperhidrosis, a symptom of autonomic dysfunction, which turned out to be a relapse of anti-leucine-rich glioma-inactivated 1 (LGI1) antibody encephalitis. This case adds to the existing literature on cases of anti-LGI-1 encephalitis, a rare form of autoimmune encephalitis, and its varied clinical manifestations. It serves as a reminder to consider a wide range of differentials in patients who present with a seemingly nonspecific complaint such as excessive sweating.

## Introduction

Hyperhidrosis refers to excessive sweat production. It is classified as primary or secondary hyperhidrosis. Secondary hyperhidrosis may be a manifestation of underlying pathological states. This includes infections, not limited to bacterial, tuberculosis, or viral causes such as human immunodeficiency virus (HIV). It may be seen in endocrinology disorders such as hyperthyroidism, pheochromocytoma [1], or carcinoid syndrome. Malignancies such as lymphoma or myeloproliferative disorders may also present similarly. Drugs contributing to sweating include hypoglycemic agents, beta blockers, and less commonly, anti-depressants, cholinergic agents, or hormonal agents [2].

Hyperhidrosis has also been described in various neurological conditions including post-stroke [3], post-spinal cord injury, neurodegenerative diseases like Parkinson’s disease [4], multiple sclerosis [5], and Guillain-Barre syndrome [6]. Its pathophysiology involves a dysfunction in the regulation of the autonomic nervous system from an insult, leading to hyperactivity of the sympathetic nervous system [7]. 

The approach to a patient with excessive sweating includes history taking to characterize the sweating - focal or generalized, time of occurrence, frequency, and severity of episodes. Features suggestive of secondary hyperhidrosis include generalized or asymmetrical sweating, or sweating which occurs at any time of the day including nighttime. Physicians should look for symptoms of the aforementioned underlying disorders.

Physicians should screen for symptoms of autonomic dysfunction as a potential cause of hyperhidrosis. These include symptoms across multiple systems: cardiovascular (e.g., postural giddiness, palpitations, or syncope due to postural hypotension); gastrointestinal (e.g., changes in bowel habits, or early satiety from gastroparesis); genitourinary (e.g., bladder dysfunction, urinary retention, or impotence); and sudomotor symptoms (e.g., increased or decreased production of saliva, tears, or sweating). Relevant physical features include temperature changes, heart rate variability, postural blood pressure changes, and a full neurological examination.

## Case presentation

A 75-year-old gentleman presented to the emergency department complaining of a new onset of excessive sweating for two weeks. His medical history included coronary artery disease, empty sella syndrome complicated by hypothyroidism and hypogonadism, type 2 diabetes mellitus, hypertension, and asthma. He was also diagnosed with anti-LGI1 encephalitis three years ago when he presented with impaired memory and disorientation, followed by generalized tonic-clonic seizures. The diagnosis was confirmed via a positive serum anti-leucine-rich glioma-inactivated (anti-LGI1) antibody. He made a full recovery after completing treatment with intravenous (IV) methylprednisolone, immunoglobulin (IG), and Rituximab. Subsequently, oral steroids and anti-epileptic medications were stopped, and he remained in remission for the next two years.

Currently, he described his symptoms as *feeling cold* in his hands and feet, followed by a *warm sensation* with *sweats drenching through his clothes*. Each episode lasted a few hours, occurring every evening for the past two weeks. There was no fever, giddiness, palpitations, chest pain, or breathlessness. He had no tremors, heat intolerance, diarrhea, headache, loss of consciousness, or preceding auras. He described the experience to be different from his previous episodes of seizures. He had no neurological symptoms, including weakness, numbness, blurring of vision, dysphasia, or dysarthria. His sleep was not disrupted apart from episodes of sweating at night. He denied feeling anxious and had no recent stressors. He had a mild loss of appetite but no noticeable weight loss. He denied alcohol use or substance abuse. There was no recent travel history of note or any change in his long-term medications. 

On physical examination, he appeared sweaty, but not anxious. Despite being in a cool environment, his shirt was drenched in sweat. His temperature was normal at 36.5 °C, systolic blood pressure ranged from 140-160 mmHg without any antihypertensive medication. Heart rate was between 90 and 110 during episodes of sweating, and he had one episode of asymptomatic postural hypotension during the hospitalization. Heart, lung, and abdominal examinations were unremarkable. Pupils were equal and reactive, and he had no nystagmus. Power examination was full over all four limbs with normal tone and no sensory deficits. There were no clinical features of thyrotoxicosis. He was admitted to the general medicine ward for further workup.

Initial laboratory investigations, including full blood count, electrolytes, cardiac enzymes, liver and renal panel, and thyroid function test, were unyielding. C-reactive protein and procalcitonin were normal. Capillary blood glucose taken during the episode of sweating did not reveal hypoglycemia. A bedside electrocardiogram was unremarkable for arrhythmias. 

Given the persistence of symptoms, a computed tomography scan of the thorax, abdomen, and pelvis was done to rule out occult infections or malignancies such as lymphoproliferative disorders. This returned negative. A 24-hour urinary catecholamines, HIV screen, and TB Quantiferon assay were negative as well. Given his background history of empty sella syndrome, a full hormonal panel, including testosterone and 8 am cortisol levels, was performed, but the results were again unremarkable. He was evaluated by the psychiatry team to rule out an underlying anxiety disorder, which could either cause or result from excessive sweating. He was prescribed alprazolam tablet on an as-needed basis to treat for possible panic disorder but with no improvement in his symptoms. 

He continued to experience repeated episodes of paroxysmal sweating with no accompanying symptoms. Apart from the single episode of asymptomatic postural hypotension, there were no other autonomic symptoms such as dry mouth, constipation, urinary retention, or erectile dysfunction. 

The neurology team was consulted at this point to consider possible autonomic dysfunction causing his symptoms of sweating. Given his previous history of anti-LGI1 encephalitis, and having ruled out other sinister causes of his sweating, the patient was treated for a possible relapse of his anti-LGI1 encephalitis. He was treated with IV methylprednisolone at 1 g/day for five days, along with IVIG at 2 g/kg over five days. MRI of the brain with contrast showed no abnormal parenchymal or meningeal enhancement, and no evidence of acute infarction, hemorrhage, or mass. A formal autonomic nervous system study was conducted, revealing absent sympathetic skin reflexes in all four limbs, which suggests underlying sudomotor dysautonomia. Electroencephalogram monitoring was not performed as an epileptic event was unlikely, and his current episode differed from his past seizure morphology. A repeat antibody testing for serum LGI1 antibody was not performed in view that this was a relapse.

Upon completion of a course of steroids and IG, our patient reported a quick and overall improvement in his symptoms, with fewer episodes of sweating. He was subsequently discharged with a neurology follow-up. Three months post-discharge, the patient reported no changes in cognition, no memory difficulties, and no recurrence of sweating. He was able to return to his activities of daily living.

## Discussion

Our patient presented with nonspecific complaints of excessive sweating, with no clear accompanying symptoms. He was well-examined in between the episodes of sweating apart from occasional hypertension, tachycardia, and an episode of postural hypotension. He had unremarkable initial investigations, and the workup for occult infections, malignancies, and endocrinological disorders was unyielding. The working diagnosis was possible anxiety or panic disorder; however, his symptoms persisted despite treatment. An eventual diagnosis of relapse of anti-LGI1 encephalitis was made after the exclusion of other causes. He responded to empirical treatment with methylprednisolone and IVIG.

Anti-LGI1 encephalitis is a rare form of autoimmune encephalitis. Typical presentations include seizures, typically faciobrachial dystonic seizures, cognitive dysfunction, rapidly progressive dementia, or personality change [8,9]. Symptoms of autonomic dysfunction are less well-documented in the literature, with only a few case reports available. One case reported excessive sweating and salivation, attributed to the effects of antibodies on post-ganglionic sympathetic neurons [10]. Another case reported paroxysmal hyperhidrosis over one side of the body [11], and postural hypotension was attributed to immune-mediated central autonomic dysfunction [12]. MRI of the brain may report hyperintensity in the medial temporal lobe or hippocampus. Diagnosis requires detection of autoantibody in serum or cerebrospinal fluid. Treatment includes high-dose steroids, IVIG, and other immunosuppressants as well as plasmapheresis [13]. Second-line immunotherapy is initiated only in patients who fail to respond or deteriorate after first-line treatment [14].

## Conclusions

Our patient had an atypical presentation of acute hyperhidrosis, uncovering an underlying autonomic dysfunction, leading to the diagnosis of a relapse of his anti-LGI1 encephalitis. The current presentation was also different from his initial manifestation of seizures. There were no abnormalities in biochemical or imaging studies; hence, the diagnosis of relapse of anti-LGI1 encephalitis was delayed. Hyperhidrosis is an underrecognized manifestation of potentially serious pathologies. This case highlights the importance of thorough history taking and physical examination in the evaluation of patients with nonspecific complaints such as sweating. A delay in the diagnosis can lead to poor treatment outcomes.
